# Evidence for the hallmarks of human aging in replicatively aging yeast

**DOI:** 10.15698/mic2016.07.510

**Published:** 2016-06-20

**Authors:** Georges E. Janssens, Liesbeth M. Veenhoff

**Affiliations:** 1 European Research Institute for the Biology of Ageing, University of Groningen, University Medical Centre Groningen, Antonius Deusinglaan 1, 9713 AV, Groningen, The Netherlands.

**Keywords:** hallmarks of aging, human, replicative aging, yeast

## Abstract

Recently, efforts have been made to characterize the hallmarks that accompany and contribute to the phenomenon of aging, as most relevant for humans 1. Remarkably, studying the finite lifespan of the single cell eukaryote budding yeast (recently reviewed in 2 and 3) has been paramount for our understanding of aging. Here, we compile observations from literature over the past decades of research on replicatively aging yeast to highlight how the hallmarks of aging in humans are present in yeast. We find strong evidence for the majority of these, and summarize how yeast aging is especially characterized by the hallmarks of genomic instability, epigenetic alterations, loss of proteostasis, deregulated nutrient sensing, and mitochondrial dysfunction.

## INTRODUCTION

**Figure 1:**
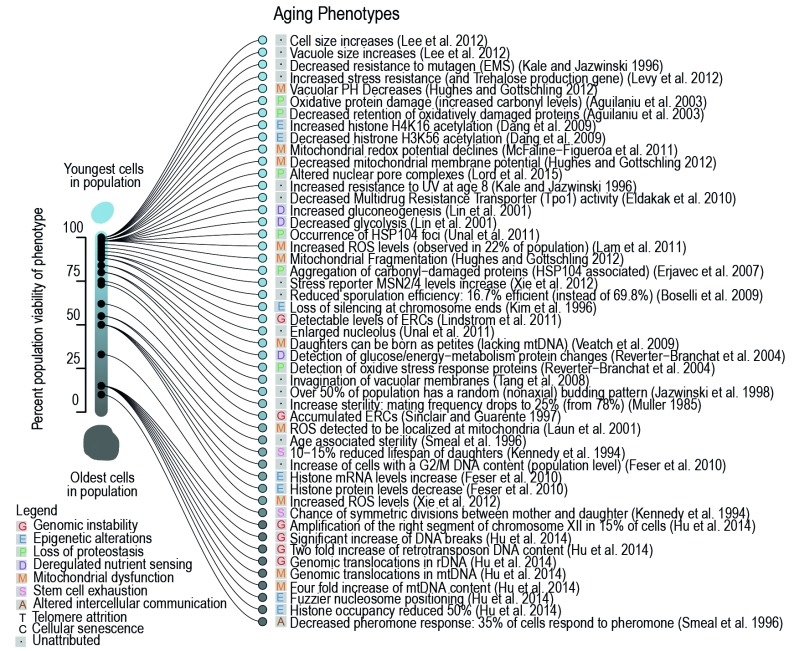
FIGURE 1: Phenotypes occurring in the yeast replicative lifespan. Observations collected from primary literature as discussed herein and as associated to a relevant hallmark of aging (symbol in legend). Cited references are often not the first to observe the phenotype but are selected based on the earliest most precise report of the timing within the RLS (i.e. while 23,24 described aged cells to be larger than young, 25 clearly observed this increase in size for a specific age, namely from the beginning on, when 100% of the population was still viable). Percentage of viability, at which age-related phenotype occurs in the population, was either directly taken from the primary literature source, or inferred based on the age of the yeast used in the publication, its average lifespan (if provided), or the average lifespan estimated from a compiled lifespan curve.

The field of the biology of aging has benefited immensely from studying the finite lifespan of the budding yeast *Saccharomyces cerevisiae* (recently reviewed in 2 and 3). Aging can be assessed in multiple ways in this single-celled eukaryote, the most common of which are by measuring the limited number of replications it can undergo 4, or by measuring the chronological time it can spend in a non-dividing state before losing viability 5. Termed the replicative lifespan (RLS) and chronological lifespan (CLS), respectively, overlap and differences exist between the factors affecting lifespan in the two models of aging 6. Our review here will focus on the RLS model of aging.

**Figure 2:**
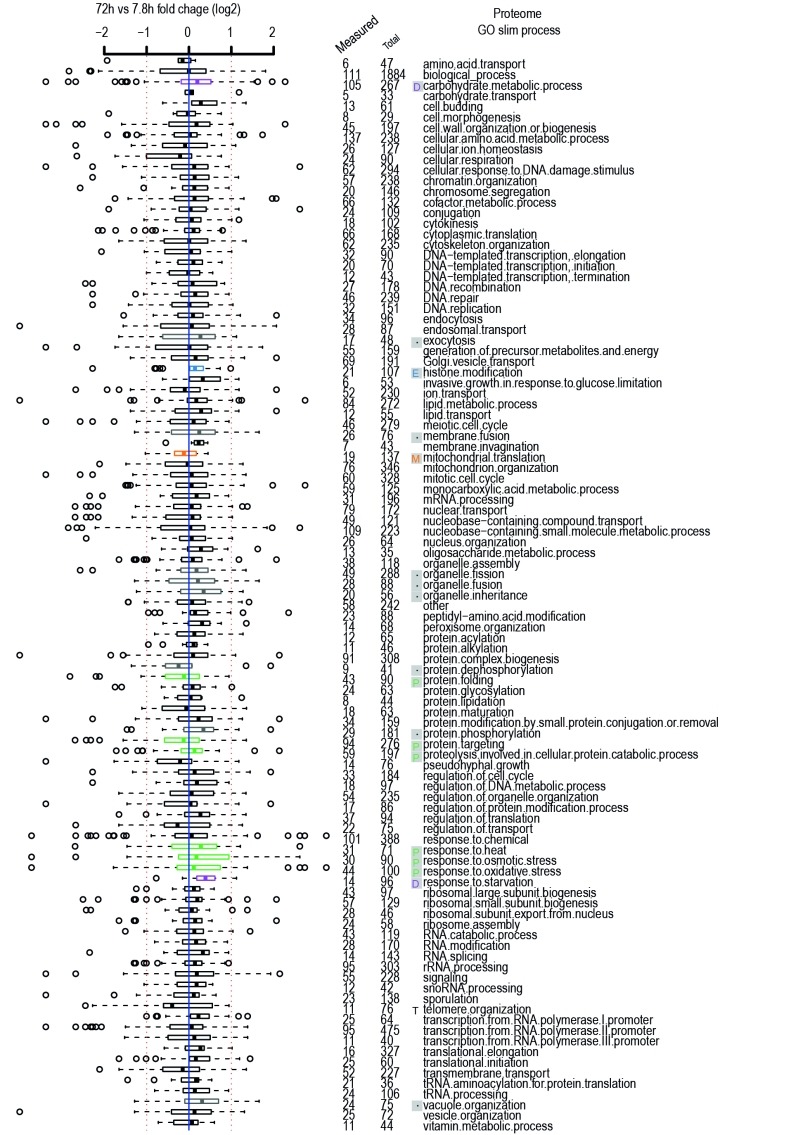
FIGURE 2: Age-related changes in Gene Ontology Processes. Gene ontology process categories taken from GO Slim lists of yeastgenome.org. Relevant Hallmarks of Aging are listed next to terms, as discussed in the text, same legend as Figure 1. Plot is comparison of old (~72 hours of aging with roughly 42% viability of the population) versus young (~8 hours of aging with nearly 100% viability of the population) protein-level changes from the 7 dataset. Data expressed in terms of fold change on a log2 scale. Red dashed line indicates two fold change. Blue solid line zero change. Numbers of proteins measured per GO term are listed out of total proteins in category. Distributions of fold changes per GO term expressed as horizontal box-and-whisker plots. The thick black line within the box is the median of the data, the box extends to the upper and lower quartile of the dataset (i.e. to include 25% of the data above and below the median, respectively), whiskers (dashed lines) represent up to 1.5 times the upper or lower quartiles and circles represent outliers.

Recently, an effort has been made to characterize the hallmarks that accompany aging 1 as most applicable to humans. Defined as the time-dependent functional decline affecting living organisms, nine hallmarks were designated as being contributors to the aging process 1. These hallmarks are: genomic instability, telomere attrition, epigenetic alterations, loss of proteostasis, deregulated nutrient sensing, mitochondrial dysfunction, cellular senescence, stem cell exhaustion, and altered intercellular communication 1. The purpose of our review is to place the aging related changes occurring in yeast within the context of these hallmarks of aging from higher eukaryotes, by collecting and organizing relevant literature (Table 1, Figure 1), and exploring pathways that change with age from proteome data resulting from our recent survey of aging yeast 7 (Figure 2). A yeast cell begins its life emerging as a bud from a mother cell. It has had its age reset, rejuvenated when compared to its mother. Soon after separation from the mother cell, it begins to produce daughters of its own. Now also a ‘mother,’ it will undergo a limited number of divisions, which is a model for aging of mitotically active cells in higher eukaryotes 2. The entirety of this process essentially reflects the hallmarks of aging for stem cell exhaustion and to some degree 8,15 cellular senescence in humans. These hallmarks however are intended to capture the decline in regenerative potential of tissues that occurs with aging 1, considered to be in fact partially affected by cell extrinsic factors 16, and we will not further elaborate on these. Several other hallmarks of human aging will not be discussed in detail here, because they have limited relevance for natural aging in yeast, or they have not been robustly implicated in yeast aging. For example, though telomeres have been implicated in yeast aging (17,18, Table 1), no significant telomere attrition occurs while the mother cell is replicating 19. Meanwhile, for altered intercellular communication, signs are present of its involvement relevant to yeast aging but only in limited form 20,21,22. Rather, much more compelling evidence exists for the hallmarks of genomic instability, epigenetic alterations, loss of proteostasis, deregulated nutrient sensing, and mitochondrial dysfunction in the yeast replicative lifespan (Figure 1), which are described in greater detail below.

**Table 1:** Overview of system wide gene expression studies in replicatively aging yeast. Publications covering either transcriptomes (first 8 rows) or proteome (last row) of wild-type aging yeast. General terms of increasing or decreasing changes are taken as described in the original publications. *Ages inferred from hours of cultivation, assuming a two-hour division time. Color code and superscripts associate changes reported in yeast relative to the hallmarks of human aging as seen in Figure 1 and described in the text; ‘G’ genomic instability, ‘E’ epigenetic alterations , ‘P’ loss of proteostasis , ‘D’ deregulated nutrient sensing , ‘M’ mitochondrial dysfunction .

**Publication**	**Time points**	**Replicative Ages**	**Increased Expression**	**Decreased Expression**
Lin *et al.* 2001 12	2	0; 7-8	Gluconeogenesis ^D^, energy storage^ D^	Glycolysis ^D^
Lesur *et al.* 2004 9	2	0; 18	Gluconeogenesis ^D^, glyoxylate cycle , lipid metabolism, glycogen production^ D^, stress response (from DNA damage signature set)^ G^	Ribosomes
Koc *et al*. 2004 11	3	0; 8–12; 8-24	Transport related genes	Glycolysis^ D^, and protein synthesis, folding (chaperones), and degradation (proteasome subunits)^ P^
Laun *et al*. 2005 8	2	0; 15 (senescent)	DNA damage response (dsDNA break repair)^ G^, cellular stress response, mitochondrial components^ M^, lipid metabolism, cell wall restructuring.	
Yiu *et al*. 2008 13	4	0; 8; 12;18-20	Aerobic metabolism, environmental stress response, nucleotide excision repair, regulatory subunits of Glc7	Ribosomes, nucleolus, methylation related metabolism
Hu *et al*. 2014 10	2	0; 26-30	All transcripts increased on average 1.3 fold due to loss of silencing, especially transposable elements and genes in rDNA locus^ E^	H3 histone (protein level)
Kamei *et al*. 2014 14	4	1; 4; 7; 11	Sugar metabolism^ D^, TCA cycle^ D^, amino acid degradation	Amino acid biosynthetic pathways
Janssens & Meinema *et al*. 2015; transcriptome 7	12	4; 5; 7; 9; 11; 13; 16; 19; 23; 27; 31; 36 *	Gene products integral to membrane, cell wall, sporulation, sterol biosynthesis, stress response (general)	Mitochondria (translation and respiration)^ M^, amino acid biosynthesis, retrotransposons, translation regulation, ribosomes, polarized growth, cortical actin cytoskeleton, ATP/GTP binding, tRNA synthesis
Janssens & Meinema *et al*. 2015; proteome 7	12	4; 5; 7; 9; 11; 13; 16; 19; 23; 27; 31; 36 *	Glycolysis/gluconeogenesis^ D^, energy reserves^ D^, stress response (general), cell wall, ATP/GTP binding, carbohydrate transport, oxidation reduction, translation regulation	Mitochondria (general, ETC, membrane)^ M^, stress response (osmotic), DNA replication ^G^, intracellular signaling, cytoskeleton, unfolded protein binding^ P^

## GENOMIC INSTABILITY

Genomic instability, defined simply as unintended alterations occurring in the genome 26, has been hypothesized to be central to aging for over half a century, as exemplified by the proposal of the Somatic Mutation Theory of Aging 27,28. This has been supported by evidence that the integrity of DNA is constantly challenged by chemical, physical, and biological agents that react with DNA 29, and that somatic mutations accumulate in cells of aging humans and model organisms 30. Furthermore, the fact that defects in the DNA repair machinery cause diseases of accelerated aging such as Werner and Bloom syndromes 31, has earned genomic instability the title of being a hallmark of aging.

In yeast, genomic instability has been a subject of extensive research due to its relevance to cancer 32. In relation to aging, *SGS1*, which encodes a RecQ helicase and is homologous to the genes mutated in Werner and Bloom syndromes in humans, shortens lifespan drastically when removed 33. Likewise, removing other proteins in the DNA damage response pathway, such as the 5' to 3' exonuclease and 5' flap endonuclease Rad27 and the single-stranded DNA-dependent ATPase, ATP-dependent nuclease, and helicase Dna2, has been shown to result in decreased lifespan for the cell 34. At the transcriptional level, a signature of DNA damage has been described to occur during aging 9 (Table 1), indicating the presence of insults to the genome, and furthermore, diploid yeast have been shown to lose heterozygosity with aging 35, likely due to compromised maintenance of genome integrity 35,36.

Although responses to DNA instability seem to exist in aging yeast cells, it has been widely accepted that the accumulation of mutations is not a cause of aging for yeast 2. This is clear from the observation that while daughters of advanced age mothers are born aged, their granddaughters are born rejuvenated, indicating that the genome has remained intact and operable 37. Therefore, observations of genetic changes such as increased transposable element activity and amplification of chromosome arms are thought only to occur in extreme late life 10. Through recent genome sequencing searching for mutations in aging yeast, evidence was found that the integrity of the genome is preserved during replication, and the accumulation of mutations is not considered to be a cause of aging in yeast 38.

A more relevant interpretation of aging related genomic instability in yeast could be related to extrachromosomal rDNA circles (ERCs) 39. ERCs are circles of DNA from the rDNA region of the genome, which may loop out by homologous recombination after attempts occur to repair a nearby double strand break 39. Once formed, the presence of an origin of replication on the ERC causes its replication during S-phase of the cell cycle 39. With a biased retention of ERCs in the mother cell, these circles of DNA accumulate with replicative age 39. While the exact mechanism of how ERCs are asymmetrically retained in aging cells remains debated (see 2 for a discussion), what is clear is their influence on aging: reducing their formation results in increased lifespan while artificially increasing their formation results in shorter lifespan 39,40. Indeed, accumulation of any self-replicating circular DNA has been shown to decrease lifespan in yeast 41. Furthermore, it has been proposed that genomic stability in the rDNA locus is the mechanism by which mutants that decrease ERC formation, increase lifespan, supporting an ‘rDNA-theory of aging’ 42,43,44,45,46. Finally, it has been shown that rDNA undergoes genomic translocations with aging 10, a sign of genomic instability.

It has been proposed that the accumulation of ERCs burden the normal replication machinery over time, compromising the cell’s ability to replicate genomic DNA, thereby limiting lifespan 39. This has been supported by the observation that a yeast mutant over-amplifying 2μ DNA circles possesses a relatively longer S-phase 47. Though the exact mechanism by which the instability at the rDNA locus limits lifespan is not fully known, it is well established that ERCs are a result of instability in the genome 43,44 and it is therefore clear that the hallmark of genomic instability is a potent factor in the replicative lifespan of yeast.

## EPIGENETIC ALTERATIONS

Epigenetic alterations of the genome, such as those of DNA methylation patterns, posttranslational histone modifications, and chromatin remodeling, have been termed a hallmark of aging 1. For example in mammals, increased trimethylation of histones H4K20 (seen in rat liver 48) and H3K4 (seen in neurons from human prefrontal cortex 49), have been reported to occur (reviewed in 50 and 51 along with epigenetic marks in model organisms). Adding to this, modification of certain components of histone modifying complexes extends lifespan in worms and flies 52,53,54. Perhaps most convincingly for its relevance to humans, the DNA methylation state of blood cells was recently shown to have predictive ability on all-cause mortality in later life 55,56.

In yeast, epigenetic alterations have been shown to have a strong influence on lifespan, especially when considering changes in histones. Perhaps most notably, the histone deacetylase Sir2 has been shown to have lifespan extending effects when overexpressed in yeast 57, as in worms and flies 58. Furthermore, increased histone H4K16 acetylation and decreased H3K56 acetylation has been observed to occur with aging 59. This age-related increase of H4K16 acetylation is thought to be related to the natural decline in levels of Sir2 that occurs with aging 59, which is responsible for deacetylation of H4K16 60. Additionally, specific gene deletions in the SAGA/SLIK (Spt-Ada-Gcn5-Acetyltransferase)/(SAGA-Like) histone deubiquitinase modules have been shown to increase lifespan in a Sir2-dependent manner 61. Furthermore, histone protein levels themselves have been shown to go down with aging 62. This decline in abundance of histones clearly has a direct effect on the lifespan of the cells, since counteracting this loss of histone levels has been shown to increase lifespan in a manner seemingly independent from other longevity pathways 62.

Overall, we find evidence for aging-related changes in epigenetic control systems in yeast (Figure 1, Figure 2). This hallmark of aging seems to manifest itself mainly at the level of histone modifiers and the histones themselves (in yeast there is no known role for cytosine DNA methylation in aging since its presence in the genome is generally thought to be absent 63), where increased histone production or increased activity of certain histone modifiers, can increase lifespan 57,61,62.

## LOSS OF PROTEOSTASIS

Protein homeostasis (proteostasis), as it pertains to aging, is generally enforced in the cell through regulation of protein production, autophagy, proteasomal degradation, and chaperone-mediated protein folding 1. Inactivation or inefficiency of these systems results in degenerative effects, such as the formation of protein aggregations, which are generally thought of as being contributors to aging 1. Aging-related disorders have been linked to impaired protein homeostasis, including Alzheimer's, Parkinson's and Huntington's 64, and many studies have shown proteostasis to change with age 65, such as having a reduced activity in stress-induced protein chaperones 66. Circumventing this decline by increasing chaperone protein levels has repeatedly been shown to increase longevity in worms and flies 67,68,69,70. Recently, mice with increased autophagy levels induced by overexpression of the autophagy related protein *ATG5* showed a 17% increase of lifespan 71. Taken together, the natural decline in pathways promoting proteostasis and the influence these can have on longevity when upregulated, has earned proteostasis its rank as a hallmark of aging.

In yeast, to see if similar decline in chaperones occurs with aging, we can look at relevant gene ontology classes in the yeast aging proteome (Figure 2) and transcriptomes (Table 1). When observing the proteins measured for protein folding and protein targeting, we find that a slight decline also occurs with age 7 (Figure 2), and likewise decreased levels of protein folding chaperones have been described at the transcriptional level (Table 1, 11). Contrary to this, however, a strong induction of specific chaperone proteins occurs with age, as these are part of the ‘response to stress’ gene ontology seen to be enriched with age (Table 1, 7,8). This increase in chaperone proteins has perhaps best been illustrated by single cell studies using fluorescent reporters tracking the increased abundance levels of the protein chaperone Hsp104 72,73. The specific induction of chaperones that occurs with aging may reflect a targeted stress response the cell is activating, and indeed the gene ontologies for response to heat, response to osmotic stress, and response to oxidative stress (31, 30, and 44 proteins measured respectively, with partial overlap) have their proteins generally increase with age 7 (Figure 2).

Damage to proteins in yeast has been previously described to occur with aging in the form of increased carbonyl levels from oxidative damage 74,75. With this, an aggregation of chaperones, namely the heat shock protein Hsp104, has been reported to occur with aging 76,77. However, neither deletion nor up-regulation of the protein chaperone *HSP104* gene has been reported to have a significant effect on lifespan 78,79, suggesting that it may indeed be the case that the cell mounts a sufficient response to sequester damaged proteins, and that the cell is not near to attaining the maximal capacity of its cytosolic protein chaperones.

Nonetheless, increasing protein homeostasis through an induction of the proteasome rather than chaperone activity has been demonstrated to have one of the strongest positive effects on lifespan reported in yeast 80. This perhaps reflects an increased age-related need for proteostasis at the level of protein turnover rather than chaperone activity, and indeed, proteolysis involved in cellular protein catabolic processes slightly increases with aging 7 (Figure 2). Recently, we have shown that a loss of stoichiometry in certain protein complexes occurs with aging 7. For example, we have noted that the nuclear pore complex (NPC) loses stoichiometry of its components with age 7, which is supported by other observations in literature that levels of the NPC components Nup116 and Nsp1 decrease relative to other components of the complex 81. Resetting the imbalance of protein complexes that naturally occurs with aging may be one of the mechanisms by which cells slow down aging through increased proteasome activity. Further work is required to understand the exact mechanism by which increased proteasome activity increases lifespan, and the hallmark of proteostasis remains a potent modulator of yeast lifespan.

## DEREGULATED NUTRIENT SENSING

Several nutrient sensing pathways have been robustly implicated in aging across multiple species 1. These include the ‘target of rapamycin’ protein kinase TOR, which senses high amino acid concentrations (reflecting high-energy availability for the cells); the AMP activated protein kinase AMPK, which detects high AMP levels (reflecting low-energy availability for the cells); and the histone modifying sirtuins, which detect high NAD^+^ levels (reflecting low-energy availability for the cells) 1. Up-regulation of the pathways sensing low-energy availability and down regulation of the pathway sensing high-energy availability results in lifespan extension 1. Longevity benefits of some of these interventions may be mediated by the downstream forkhead box transcription factor FOXO, which activates gene transcription conferring proteotoxic stress resistance 1. Furthermore, for multicellular organisms, the pathways of growth hormone, and the insulin and insulin-like growth factor 1 signaling, lie upstream in the nutrient sensing pathway, and both of these have been robustly shown to modulate lifespan across species 1.

In yeast, changes in carbon metabolism have been repeatedly described to accompany aging, and include an increase in gluconeogenesis and energy storage, and a decrease in glycolysis 9,11,12 (Table 1, Figure 2). It has been suggested that the aging yeast cell, which is continuously growing in size 82, may interpret its changing cell size to volume ratio as a cue of starvation, which in turn induces these metabolic changes 13,14. Supporting this, when looking at aging proteome data and the 14 proteins measured in the gene ontology for response to starvation, we see a general increase in abundances to occur with aging (Figure 2). Indeed, in our recently completed analysis showing causal and responsive elements in aging, metabolic changes have been found to be downstream of earlier occurring changes 7. Interestingly, while the cell may be interpreting cues of starvation, it remains on a trajectory of proliferation and continuous growth in cell size 25,82.

We previously showed that the proteome of aging yeast uncouples from the transcriptome, especially through an over-abundance of translation machinery related proteins 7. Following this, through analysis of a high-level directional network of the aging transcriptome, we found that these translation machinery genes were a causal force, shaping the behavior of other molecular changes occurring with aging 7. These observations suggest that the translation machinery and pathways for cellular growth are strong determinants of lifespan, and are suggestive of an aging-related hyperactivity of the nutrient sensor TOR, a regulator of translation machinery production. In line with this, deletions of genes encoding TOR1, the downstream protein kinase SCH9, and certain ribosomal proteins, increases lifespan in yeast 83,84,85. Likewise, increasing levels of FOXO transcription factors, which is thought to confer some of the longevity benefits associated to modified nutrient sensing pathways in higher organisms 1, increases lifespan in yeast 86.

Nutrient sensing pathways play a role in regulating protein abundance via controlling both production and degradation of proteins. What results from this is that the hallmark of aging of deregulated nutrient sensing is closely linked to the hallmark of proteostasis. Indeed, the translation machinery, which is normally regulated by nutrient sensors, seems to be at the heart of cellular aging 7,85. This, in addition to the fact that nutrient sensing pathways can modulate aging, and that clear signs of metabolic changes occur in aging yeast (Table 1), demonstrates that also in yeast, deregulated nutrient sensing is a hallmark of aging.

## MITOCHONDRIAL DYSFUNCTION

Mitochondria have been studied in the context of aging for over half a century, having gained popularity in the field with the introduction of the free radical theory of aging 87. The efficiency of the mitochondrial respiratory chain decreases with aging, reducing the membrane potential and ATP generation, and increasing electron leakage and production of reactive oxygen species (ROS) 1,88. Although once thought to be purely detrimental, we now know that low levels of ROS may induce a protective homeostatic response, while only higher levels, which may increase for a variety of reasons, accelerate aging 1. Many other changes have been proposed to contribute to aging related mitochondrial dysfunction, such as an accumulation of mutations and deletions in mtDNA, the oxidation and destabilization of mitochondrial protein complexes, a reduced biosynthesis of iron-sulfur clusters, a decline in the levels of mitochondrial biogenesis, an imbalance of fission and fusion events, and a diminishing quality control from mitophagy 89,90. The long list of factors that are thought to contribute to increased damage and decreased turnover for mitochondria have been speculated to influence the natural aging process 1, and mitochondrial dysfunction has therefore been deemed a hallmark of aging.

In yeast, an early age decline in mitochondrial membrane potential has been described at ages where normally still more than 98% of the population are viable 91,92 (Figure 1). This has been accompanied by the observation that mitochondrial fragmentation occurs with aging 91,93, and decreased amounts of certain mitochondrial components are detected in aged cells 7 (Table 1, Figure 2). An aging related decline of vacuolar pH has also been associated to dysfunction of mitochondria 91. Specifically, it was observed that vacuolar acidity drops early in aging, and is followed by the previously described structural and membrane potential changes in the mitochondria. Improving the age-related decline in vacuolar pH by overexpression of a subunit (Vma1) of the vacuolar ATPase complex that establishes acidity levels, concomitantly delays mitochondrial dysfunction and extends lifespan 91. Though targeting the vacuole, this observation is the strongest to link interventions in mitochondrial biology in yeast with increased lifespan. Highlighting the heterogeneity of the aging phenotype though, a recent report has suggested that only a subset of the population is affected by loss of mitochondrial membrane potential in this way 15. A second observation linking change in mitochondria to longevity is the occurrence of petites, cells which are defective in respiration 94. The transformation of a yeast cell into a petite can result in an increased lifespan for the cell under certain, but not all, conditions 95. Interestingly, an increase in the rate of formation of petite daughter cells may be occurring during aging 94, which could coincide with observations that mtDNA translocate to the nuclear genome in mother cells of advanced age 10.

Mitochondrial malfunction has the potential to cause detrimental effects in the yeast cell. Increased ROS levels have been observed to occur with aging 72, as have levels of ROS specifically localized to the mitochondria 96. This may explain why oxidative damage to proteins increases markedly in aging yeast 75,77, and why a strong response to oxidative damage and oxidative stress is induced 74,77. Overall, clear signs are present that mitochondrial dysfunction is a hallmark of yeast aging, from altered mitochondrial morphology and membrane potential, to the production of ROS and the increasing damage it causes to proteins 75,77.

## DISCUSSION

We have conducted a tour of the literature to shed light on the aging process in yeast, and have found clear signs that the majority of hallmarks of aging present in vertebrates also manifest themselves in replicatively aging yeast. Additionally, we note that other phenotypes of aging exist in yeast that may not readily classify under a specific hallmark. For example, we see that proteins in the gene ontology for protein phosphorylation and protein dephosphorylation appear to undergo opposing abundance changes with age 7 (Figure 2), suggesting an overall increase in phosphorylation state. The pathways of endocytosis, membrane fusion, and organelle fission, fusion and inheritance are all seen to undergo diverse changes with age at the protein level 7 (Figure 2), pointing towards a potentially important role in aging. Also, the yeast vacuole seems greatly affected as proteins involved in its organization increase with age 7 (Figure 2), certain vacuolar proteins preferentially accumulate in mother cells 97, and the vacuole enlarges with signs of invagination 25,98. Other changes occurring in aging yeast include a reduction in sporulation efficiency 99, an increasingly random budding pattern 100, an increase in sterility 20, an enlarging nucleolus and increased G2/M DNA content 62,76, and either increased or decreased sensitivity to drugs, stresses, and mutagens 101,102,103 (Figure 1). Studying why and how these aging related phenotypes happen in yeast may provide interesting avenues of future research in higher organisms. Clearly, there is still more to be gained from yeast for understanding human aging.

The hallmarks of aging as presented in 1 were chosen partially based on their ability to confer extended longevity with interventions. Likewise, in our tour of literature, we have found this to be the case for the hallmarks of human aging that are applicable to yeast. For each hallmark, specific interventions can extend lifespan of yeast. For genomic instability, deleting *FOB1* reduces ERC formation and extends longevity 40. For epigenetic modifications, increasing levels of Sir2 57, histones H3 and H4 62, or deletion of genes encoding the SAGA components *SGF73*, *SGF11*, and *UBP8* 61, results in a modified epigenome and extended longevity. For the hallmark of proteostasis, deletion of *UBR2* was shown to increase lifespan by increasing proteasome activity 80. For deregulated nutrient sensing deletions of *TOR1* or *SCH9* increase lifespan 84,85 as does dietary restriction (DR) mimetics such as the *HXK2* deletion strain 104, and DR itself under certain, but not all, conditions 104,105,106. Likewise, FOXO, thought to be downstream in many nutrient-sensing pathways that increase lifespan when modulated, increases lifespan in yeast 86. Finally, for the hallmark of mitochondrial dysfunction, improving mitochrondrial function by overexpression of the vacuolar ATPase protein Vma1 results in lifespan extension 91.

Interestingly, as depicted in Figure 1, many of the phenotypes of aging manifest themselves early in the yeast’s lifespan, while still 90% of the population remains viable. The presence of many early-age phenotypes in literature is partially due to the fact that enriching yeast cultures for cells of advanced age is a difficult process, and it is therefore easier to characterize younger ‘aged’ cells 2. Despite this, studies that have explored into deeper ages in the population’s viability have nonetheless found relatively similar changes at the process level to occur in middle and old ages (Table 1). Indeed, we previously found that most changes occur early and are progressive over time 7. Since most of the studies characterizing aging cells were done at the population level, interesting questions that arise are whether the occurrence of these phenotypes are delayed in naturally longer-lived cells within a population, if these longer lived cells may be more resistant to certain changes, or if they may have different pathologies and causes of mortality than naturally shorter lived cells. Recently, these questions have drawn the attention of researchers and are beginning to be answered 15,25,72,73,107.

As discussed in a dedicated review 23 the change in one phenotype impacts many others, leading to either the sequential collapse of different functions or the triggering of compensating responses making up for the decline of one subsystem. To begin to map these connections on a system wide level, we predicted based on network analysis that the changes in the genes related to protein biogenesis are causal to diverse other age related changes, putting the hallmark of protein homeostasis at the center of attention. Indeed, system-level phenotypes of aging in yeast 7, have been shown to be conserved in humans and rhesus macaques (brain prefrontal cortex 24) and rat (brain, liver 108) and include a progressive uncoupling of the proteome from the transcriptionally encoded message, and a change in stoichiometry of the components of certain protein complexes.

In studies more dedicated to understanding individual molecular mechanisms and pathways of aging, elegant examples of the interconnected nature of the aging process can be found. For example, one sequence of events involving the plasma membrane, vacuole, and mitochondria and linking to genome stability bridges several hallmarks of aging, as follows. The sequence starts with the activity of the plasma membrane ATPase, Pma1, which already early in life causes the cytosolic pH of yeast to increase 109. This activity antagonizes vacuole acidity by reducing cytosolic protons, which results in an increased vacuolar pH. This, in turn, compromises storage of neutral amino acids 91. An excess of these in the cytosol may overburden the mitochondrial dependent catabolism of amino acids, which could be a cause of the loss in mitochondrial membrane potential 91. A loss in mitochondrial membrane potential culminates in a reduced synthesis of iron-sulfur clusters 94, and, as these are required as cofactors in many DNA repair enzymes, the entire cascade of events thus links to genomic stability 35.

Interestingly, overexpression of only one of the component of the vacuolar proton ATPase, *VMA1*, results in lifespan extension and rescue of some of the above mentioned phenotypes 91. The vacuolar proton ATPase is one of the complexes in a yeast cell that shows a clear loss of its stoichiometry in replicative aging, and indeed the protein abundance of *VMA1* decreases relative to most other components 7. We speculate that the rescue of the aging phenotypes from overexpression of *VMA1* is thus related to the general problem of maintaining protein complex stoichiometry in aging.

In some cases feed forward loops may also present themselves, driving cell death. E.g. the nuclear pore complex shows a strong loss of the stoichiometry of its protein components 7 which may be a cause of altered transport function, impacting ribosome biogenesis, mRNA export, and import of transcriptional regulators and tRNA, all feeding back into the potential causes of the NPC sub-stoichiometry. Finding how the different hallmarks are dependent on each other remains an important task for the future to understand the aging process.

Overall, we find evidence that the hallmarks involved in vertebrate aging are implicated in the replicative aging of yeast, indicating that aging is affected by dysfunction of shared processes. Nutrient sensing and mitochondria are vital for activity of the cell, while proteostasis is integral to the maintenance of the cellular system. Meanwhile the very identity of the cell, the genome and epigenome, are essential to carry the message for what processes and functions should occur. These processes are universally necessary to support cell growth, viability and division in all eukaryotic life, from yeast to vertebrates, and it follows that their dysfunction should be integral to disease and aging.
